# PTB-XL, a large publicly available electrocardiography dataset

**DOI:** 10.1038/s41597-020-0495-6

**Published:** 2020-05-25

**Authors:** Patrick Wagner, Nils Strodthoff, Ralf-Dieter Bousseljot, Dieter Kreiseler, Fatima I. Lunze, Wojciech Samek, Tobias Schaeffter

**Affiliations:** 1 Physikalisch-Technische Bundesanstalt, Berlin, Germany; 2 Fraunhofer Heinrich Hertz Institute, Berlin, Germany; 3 Technical University Berlin, Berlin, Germany; 4 German Heart Center Berlin, Charité - Universitätsmedizin, Berlin, Germany; 5 King’s College London, London, UK

**Keywords:** Machine learning, Data publication and archiving, Electrocardiography - EKG, Cardiovascular diseases

## Abstract

Electrocardiography (ECG) is a key non-invasive diagnostic tool for cardiovascular diseases which is increasingly supported by algorithms based on machine learning. Major obstacles for the development of automatic ECG interpretation algorithms are both the lack of public datasets and well-defined benchmarking procedures to allow comparison s of different algorithms. To address these issues, we put forward *PTB-XL*, the to-date largest freely accessible clinical 12-lead ECG-waveform dataset comprising 21837 records from 18885 patients of 10 seconds length. The ECG-waveform data was annotated by up to two cardiologists as a multi-label dataset, where diagnostic labels were further aggregated into super and subclasses. The dataset covers a broad range of diagnostic classes including, in particular, a large fraction of healthy records. The combination with additional metadata on demographics, additional diagnostic statements, diagnosis likelihoods, manually annotated signal properties as well as suggested folds for splitting training and test sets turns the dataset into a rich resource for the development and the evaluation of automatic ECG interpretation algorithms.

## Abstract (summary)

**Table:** 

Measurement(s)	electrocardiography • cardiovascular system
Technology Type(s)	12 lead electrocardiography
Factor Type(s)	presence of co-occurring diseases
Sample Characteristic - Organism	Homo sapiens

Machine-accessible metadata file describing the reported data: 10.6084/m9.figshare.12098055

## Background & Summary

Cardiovascular diseases are the leading cause of mortality worldwide, which is in high-income countries only surpassed by cancer^1^. Electrocardiography (ECG) provides a key non-invasive diagnostic tool for assessing the cardiac clinical status of a patient. Advanced decision support systems based on automatic ECG interpretation algorithms promise significant assistance for the medical personnel due to the large number of ECGs that are routinely taken. However, there are at least two major obstacles that restrict the progress in this field beyond the demonstration of exceptional performance of closed-source algorithms on custom datasets with restricted access^2,3^, (1) the lack of large publicly available datasets for training and validation^4^, and (2) the lack of well-defined evaluation procedures for these algorithms. We aim to address both issues and to close this gap in the research landscape by putting forward *PTB-XL*^5^, a clinical ECG dataset of unprecedented size along with proposed folds for the evaluation of machine learning algorithms.

**Fig. 1:**
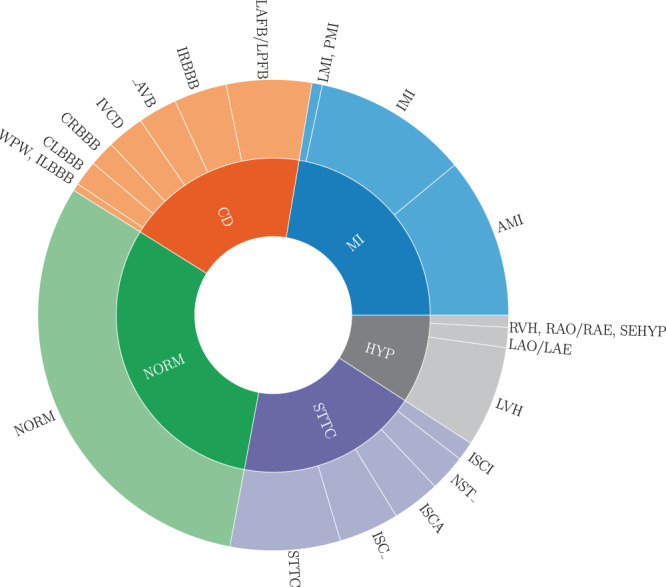
Graphical summary of the *PTB-XL* dataset in terms of diagnostic superclasses and subclasses, see Table 5 for a definition of the used acronyms.

The raw signal data underlying the *PTB-XL* dataset was recorded by devices from the *Schiller AG* between October 1989 and June 1996. The transfer of the raw data into a structured database, its curation along with the development of corresponding ECG analysis algorithms was a long term project at the Physikalisch Technische Bundesanstalt (PTB). These efforts resulted in a number of publications^6–11^, but the access to the dataset remained restricted until now. The dataset comprises $21837$ clinical 12-lead ECG records of 10 seconds length from 18885 patients. The dataset is balanced with respect to sex (52% male and 48% female) and covers the whole range of ages from 0 to 95 years (median 62 and interquantile range of 22). The ECG records were annotated by up to two cardiologists with potentially multiple ECG statements out of a set of 71 different statements conforming to the SCP-ECG standard^12^. The statements cover form, rhythm and diagnostic statements in a unified, machine-readable form. For the diagnostic labels we provide a hierarchical organization in terms of 5 coarse superclasses and 24 subclasses for the diagnostic labels, see Fig. 1 for a graphical summary of the dataset, that allow for different levels of granularity. Besides annotations in the form of ECG statements along with likelihood information for diagnostic statements, additional metadata for example in the form of manually annotated signal quality statements are available.

**Fig. 2:**
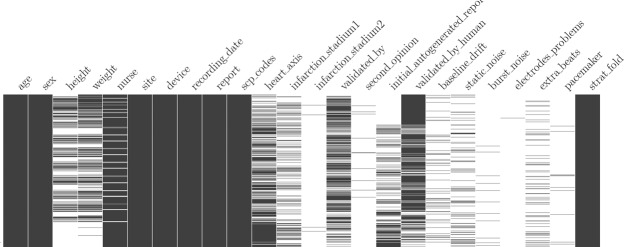
Overview of populated columns in ptbxl_database.csv. Each entry corresponds to a row in the table in temporal order from top to bottom. Black pixels indicate existing values, missing values remain white.

**Fig. 3:**
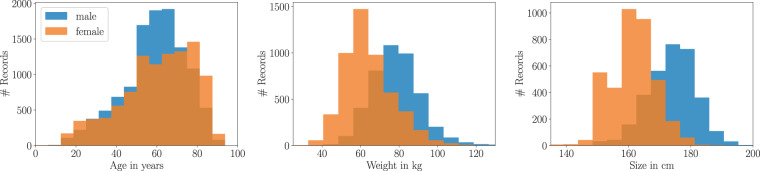
Demographic overview of patients in *PTB-XL*.

**Fig. 4:**
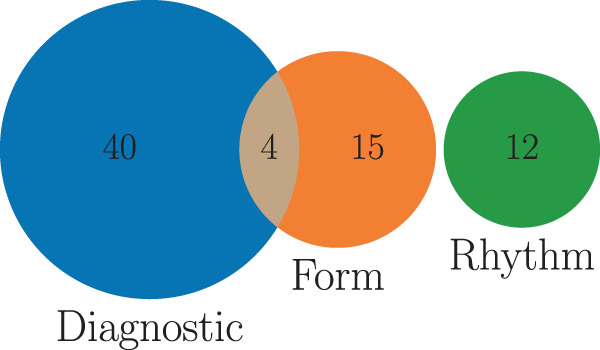
Venn Diagram illustrating the assignment of the given SCP ECG statements to the three categories *diagnostic*, *form* and *rhythm*.

**Fig. 5:**
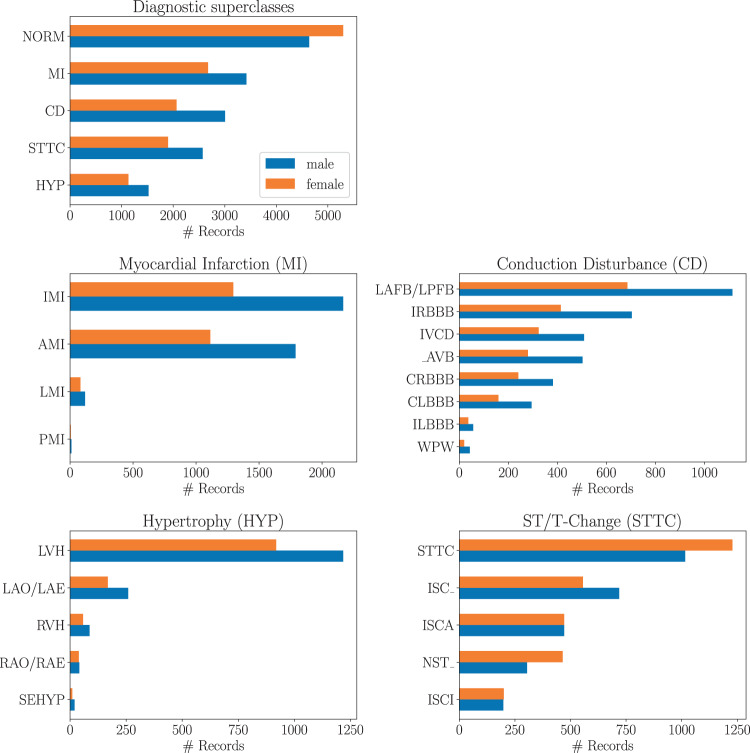
Distribution of diagnostic subclasses for given diagnostic superclasses.

**Fig. 6:**
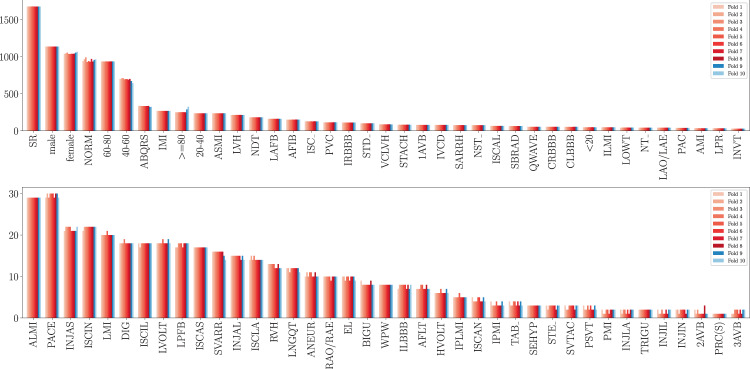
Distribution of ECG statements, sex and age across ten folds with stratified folds. The ninth and tenth fold are folds with a particularly high label quality that are supposed to be used as validation and test sets.

**Fig. 7:**
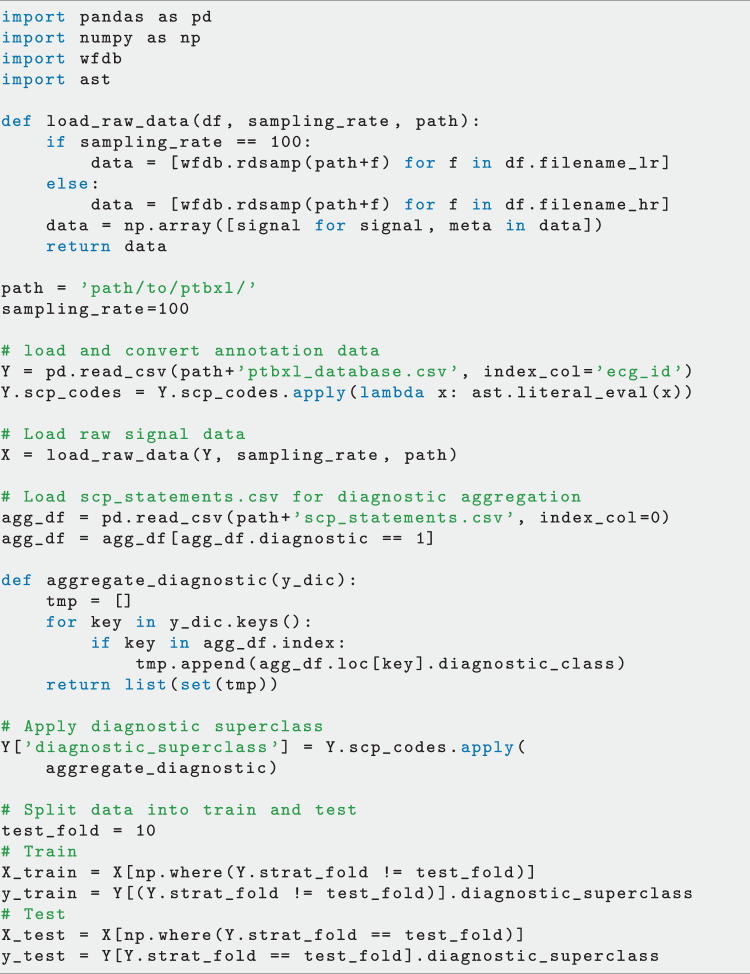
Example Python code for loading data and labels also using the suggested folds and aggregation of diagnostic labels.

Apart from the outstanding nominal size of *PTB-XL*, the dataset is distinguished by its diversity, both in terms of signal quality (with 77.01% of highest signal quality) but also in terms of a rich coverage of pathologies, many different co-occurring diseases but also a large proportion of healthy control samples that is rarely found in clinical datasets. It is in particular this diversity, which makes *PTB-XL* a rich source for the training and evaluation of algorithms in a real-world setting, where machine learning (ML) algorithms have to work reliably regardless of the recording conditions or potentially poor quality data.

To highlight the uniqueness of the *PTB-XL* dataset, we compare different commonly used ECG datasets in Table 1 based on sample statistics (number of ECG signals, number of recorded leads, number of patients, average recording length in seconds) and their respective annotations ((D)iagnostic, (F)orm, (R)hytm, (C)linical, (B)eat annotation and the respective number of classes). Most open datasets are provided by PhysioNet^13^, but typically cover only a few hundred patients. Most notably, this includes the PTB Diagnostic ECG Database^6^, which was collected during the course of the same long-term project at the PTB, which, however, shares no records with the *PTB-XL* dataset. The PTB Diagnostic ECG Database includes only 549 records from a single site and provides only a single label per record as opposed to multi-label, machine-readable annotations covering a much broader range of pathologies in *PTB-XL*. The only exceptions in terms of freely accessible datasets with larger samples sizes are the AF classification dataset^14^ and the Chinese ICBEB Challenge 2018 dataset^15^, which contain, however, either just single-lead ECGs or cover only a very limited set of ECG statements. There are several larger datasets that are either commercial or where the access is restricted by certain conditions (top five rows in Table 1). This includes commercial datasets such as CSE^16^, which has traditionally been used to benchmark ECG interpretation algorithms.

**Table 1:** Summary of selected ECG datasets.

	Name	# ECG	# Leads	# Patients	Average length in seconds	Available labels	# Classes
restricted	CSE^16^	1220	15	1220	30	D	7
	AHA^20^	154	2	154	1800	DFRB	8
	Stanford^2^	64121	1	29163	30	R	14
	CCDD^21^	179130	12	179130	30	D	378
	THEW^22^ (Chest Pain LR)	1172	12	1154	86400	CB	5
	Mayo CV^3^	649931	12	180922	10	R	2
	ICBEB Challenge 2018^15^	6877	12	6877	30	DFR	8
non-restricted	MIT-BIH Noise Stress Test^23^	15	1	15	22500	B	1
	MIT-BIH Arrhythmia^24^	48	2	47	1800	B	1
	Malignant Ventricular Arrhythmia^25^	22	2	22	1800	R	3
	Ventricular Tachyarrhythmia^26^	35	1	35	480	B	3
	European ST-T Database^27^	90	2	79	7200	F	2
	AF Classification Challenge 2017^14^	8528	1	8528	32.5	R	4
	PTB Diagnostic ECG^6^	549	15	294	60	D	9
	***PTB-XL*** (this work)	$21837$	12	$18885$	10	DFR	$71$

**Table 2:** Columns provided in the metadata table ptbxl_database.csv.

Section	Variable	Data Type	Description
Identifiers	ecg_id	integer	unique ECG identifier
	patient_id	integer	unique patient identifier
	filename_lr	string	path to waveform data (100 Hz)
	filename_hr	string	path to waveform data (500 Hz)
General Metadata	age	integer	age at recording in years (see Fig. 3 left)
	sex	categorical	sex (male 0, female 1)
	height	integer	height in centimeters (see Fig. 3 right)
	weight	integer	weight in kilograms (see Fig. 3 middle)
	nurse	categorical	involved nurse (pseudonymized)
	site	categorical	recording site (pseudonymized)
	device	categorical	recording device
	recording_date	datetime	ECG recording date and time
ECG Statements	report	string	ECG report from diagnosing cardiologist
	scp_codes	dictionary	SCP ECG statements (see Tables 6, 7 and 8)
	heart_axis	categorical	heart’s electrical axis (see Table 10)
	infarction_stadium1	categorical	infarction stadium (see Table 11)
	infarction_stadium2	categorical	second infarction stadium (see Table 11)
	validated_by	categorical	validating cardiologist (pseudonymized)
	second_opinion	boolean	flag for second (deviating) opinion
	initial_autogenerated_report	boolean	initial autogenerated report by ECG device
	validated_by_human	boolean	validated by human
Signal Metadata	baseline_drift	string	baseline drift or jump present
	static_noise	string	electric hum/static noise present
	burst_noise	string	burst noise
	electrodes_problems	string	electrodes problems
	extra_beats	string	extra beats
	pacemaker	string	pacemaker
Cross-validation Folds	strat_fold	integer	suggested stratified folds

Each ECG is identified by a unique ID (ecg_id) and comes with a number of ECG statements (scp_codes) that can be used to train a multi-label classifier that can be evaluated based on the proposed fold assignments (strat_fold).

**Table 3:** Overview of number of records per patient.

# Records	1	2	3	4	5	6	7	8	9	10
# Patients	16758	1604	348	103	43	16	5	4	3	1

**Table 4:** Likelihood statements for diagnostic statements inferred from keywords in the ECG report as introduced in ECG Statements.

Keywords	Weighting Factor (Confidence)
nicht auszuschliessen, cannot rule out, cannot be excluded	15%
möglicherweise, consider, suggest, likely	35%
wahrscheinlich, possible, maybe, probably, ablaufend, Verdacht auf	50%
Sonst, Bild	80%
Consistent with, Diagnose, Zustand nach…	100%

**Table 5:** SCP-ECG acronym descriptions for super- and subclasses.

		Acronym	SCP statement Description
Superclasses		NORM	Normal ECG
		CD	Conduction Disturbance
		MI	Myocardial Infarction
		HYP	Hypertrophy
		STTC	ST/T change
Subclasses	NORM	NORM	Normal ECG
	CD	LAFB/LPFB	left anterior/left posterior fascicular block
		IRBBB	incomplete right bundle branch block
		ILBBB	incomplete left bundle branch block
		CLBBB	complete left bundle branch block
		CRBBB	complete right bundle branch block
		_AVB	AV block
		IVCB	non-specific intraventricular conduction disturbance (block)
		WPW	Wolff-Parkinson-White syndrome
	HYP	LVH	left ventricular hypertrophy
		RHV	right ventricular hypertrophy
		LAO/LAE	left atrial overload/enlargement
		RAO/RAE	right atrial overload/enlargement
		SEHYP	septal hypertrophy
	MI	AMI	anterior myocardial infarction
		IMI	inferior myocardial infarction
		LMI	lateral myocardial infarction
		PMI	posterior myocardial infarction
	STTC	ISCA	ischemic in anterior leads
		ISCI	ischemic in inferior leads
		ISC_	non-specific ischemic
		STTC	ST-T changes
		NST_	non-specific ST changes

**Table 6:** Diagnostic Statement Overview, where the acronyms of super- and subclass are introduced in Table 5.

	# Records	Description	Superclass	Subclass
LAFB	1626	left anterior fascicular block	CD	LAFB/LPFB
IRBBB	1118	incomplete right bundle branch block	CD	IRBBB
AVB	797	first degree AV block	CD	_AVB
IVCD	789	non-specific intraventricular conduction disturbance (block)	CD	IVCD
CRBBB	542	complete right bundle branch block	CD	CRBBB
CLBBB	536	complete left bundle branch block	CD	CLBBB
LPFB	177	left posterior fascicular block	CD	LAFB/LPFB
WPW	80	Wolff-Parkinson-White syndrome	CD	WPW
ILBBB	77	incomplete left bundle branch block	CD	ILBBB
3AVB	16	third degree AV block	CD	_AVB
2AVB	14	second degree AV block	CD	_AVB
LVH	2137	left ventricular hypertrophy	HYP	LVH
LAO/LAE	427	left atrial overload/enlargement	HYP	LAO/LAE
RVH	126	right ventricular hypertrophy	HYP	RVH
RAO/RAE	99	right atrial overload/enlargement	HYP	RAO/RAE
SEHYP	30	septal hypertrophy	HYP	SEHYP
IMI	2685	inferior myocardial infarction	MI	IMI
ASMI	2363	anteroseptal myocardial infarction	MI	AMI
ILMI	479	inferolateral myocardial infarction	MI	IMI
AMI	354	anterior myocardial infarction	MI	AMI
ALMI	290	anterolateral myocardial infarction	MI	AMI
INJAS	215	subendocardial injury in anteroseptal leads	MI	AMI
LMI	201	lateral myocardial infarction	MI	LMI
INJAL	148	subendocardial injury in anterolateral leads	MI	AMI
IPLMI	51	inferoposterolateral myocardial infarction	MI	IMI
IPMI	33	inferoposterior myocardial infarction	MI	IMI
INJIN	18	subendocardial injury in inferior leads	MI	IMI
PMI	17	posterior myocardial infarction	MI	PMI
INJLA	17	subendocardial injury in lateral leads	MI	AMI
INJIL	15	subendocardial injury in inferolateral leads	MI	IMI
NORM	9528	normal ECG	NORM	NORM
NDT	1829	non-diagnostic T abnormalities	STTC	STTC
NST_	770	non-specific ST changes	STTC	NST_
DIG	181	digitalis-effect	STTC	STTC
LNGQT	118	long QT-interval	STTC	STTC
ISC_	1275	non-specific ischemic	STTC	ISC_
ISCAL	660	ischemic in anterolateral leads	STTC	ISCA
ISCIN	219	ischemic in inferior leads	STTC	ISCI
ISCIL	179	ischemic in inferolateral leads	STTC	ISCI
ISCAS	170	ischemic in anteroseptal leads	STTC	ISCA
ISCLA	142	ischemic in lateral leads	STTC	ISCA
ANEUR	104	ST-T changes compatible with ventricular aneurysm	STTC	STTC
EL	97	electrolytic disturbance or drug (former EDIS)	STTC	STTC
ISCAN	44	ischemic in anterior leads	STTC	ISCA

**Table 7:** Form Statement Overview.

	# Records	Description
NDT	1829	non-diagnostic T abnormalities
NST_	770	non-specific ST changes
DIG	181	digitalis-effect
LNGQT	118	long QT-interval
ABQRS	3327	abnormal QRS
PVC	1146	ventricular premature complex
STD_	1009	non-specific ST depression
VCLVH	875	voltage criteria (QRS) for left ventricular hypertrophy
QWAVE	548	Q waves present
LOWT	438	low amplitude T-waves
NT_	424	non-specific T-wave changes
PAC	398	atrial premature complex
LPR	340	prolonged PR interval
INVT	294	inverted T-waves
LVOLT	182	low QRS voltages in the frontal and horizontal leads
HVOLT	62	high QRS voltage
TAB_	35	T-wave abnormality
STE_	28	non-specific ST elevation
PRC(S)	10	premature complex(es)

**Table 8:** Rhythm Statement Overview.

	# Records	Description
SR	16782	sinus rhythm
AFIB	1514	atrial fibrillation
STACH	826	sinus tachycardia
SARRH	772	sinus arrhythmia
SBRAD	637	sinus bradycardia
PACE	296	normal functioning artificial pacemaker
SVARR	157	supraventricular arrhythmia
BIGU	82	bigeminal pattern (unknown origin, SV or Ventricular)
AFLT	73	atrial flutter
SVTAC	27	supraventricular tachycardia
PSVT	24	paroxysmal supraventricular tachycardia
TRIGU	20	trigeminal pattern (unknown origin, SV or Ventricular)

**Table 9:** Overview of number of statements per ECG introduced in ECG Statements.

Level	0	1	2	3	4	5	6	7	8	9
Diagnostic	407	15019	4242	1515	529	121	4	0	0	0
Diagnostic Superclass	407	16272	4079	920	159	0	0	0	0	0
Diagnostic Subclass	407	15239	4171	1439	475	102	4	0	0	0
Form	12849	6693	1672	524	90	9	0	0	0	0
Rhythm	771	20923	142	1	0	0	0	0	0	0
All	0	705	11247	5114	2597	1254	597	253	63	7

**Table 10:** Distribution of heart_axis as introduced in ECG Statements.

	Keywords	# Records
UNK	Unknown	8505
MID	Normal axis	7687
LAD	Left axis deviation	3764
ALAD	Abnormal LAD, extreme left axis deviation	1382
RAD	Right axis deviation	221
ARAD	Abnormal RAD, extreme right axis deviation	122
AXL	Horizontal axis	102
AXR	Vertical axis	51
SAG	Saggital type (S1-S2-S3 Pattern)	3

**Table 11:** Distribution of infarction stadium across the dataset as introduced in ECG Statements.

	Keyword	# Records
Stadium I	acut, early	186
Stadium I–II	acut/subacut, ablaufend	5
Stadium II	recent, subacut, bereits abgelaufen	107
Stadium II–III	subacut/chronisch	943
Stadium III	old, abgelaufen, chronisch	1045
unknown	uncertain, unknown, unbekannt	3443

Counts are cumulated from infarction_stadium and infarction_stadium2 which are only set to a value if at least one statement belongs to the superclass of Myocardial Infarction (MI).

**Table 12:** SCP-ECG statement summary.

Column	Description
acronym	SCP statement
description	short statement description
diagnostic	flag if statement is diagnostic
form	flag if statement is related to form
rhythm	flag if statement is related to rhythm
diagnostic_class	superclass for diagnostic statements
diagnostic_subclass	subclass for diagnostic statements
Statement Category	official SCP statement category
SCP-ECG Statement Description	official SCP statement description
AHA code	unique ID in the AHA standard
aECG REFID	IEEE 11073-10102 Annotated ECG (aECG) standard
CDISC Code	Controlled Terminology
DICOM Code	DICOM Tags

Description of annotation scheme stored in scp_statements.csv.

## Methods

This section covers following aspects: In *Data Acquisition*, we describe in detail the data acquisition process and in *Preprocessing* we discuss the applied preprocessing steps in order to facilitate a widespread use for training and evaluating machine learning algorithms.

### Data acquisition

The raw data acquisition was carried out as follows:

1. The waveform data was automatically trimmed to 10 seconds segments and stored in a proprietary compressed format. For all signals, we provide the standard set of 12 leads (I,II,III,aVL,aVR,aVF,V1–V6) with reference electrodes on the right arm. The original sampling frequency was 400 Hz.
2. The corresponding metadata was entered into a database by a nurse.
3. Each record was annotated as follows:
  - An initial ECG report string was generated by either:
    i. 67.13% manual interpretation by a human cardiologist
    ii. 31.2% automatic interpretation by ECG-device
      A. 4.45% validation by a human cardiologist
      B. 26.75% incomplete information on human validation
    iii. 1.67% no initial ECG report.
    In *Quality Assessment for Annotation Data (ECG Statements)*, we provide a more extensive discussion on this step.
  - The report string was converted into a standardized set of SCP-ECG statements including likelihood information for diagnostic statements.
  - The heart’s axis and the infarction stadium (if applicable) was extracted from the report.
  - A potential second validation (for first evaluation in case of a missing initial report string) was carried out by a second independent cardiologist, who was able to make changes to the ECG statements and the likelihood information directly. In most cases, the deviating opinion was also reported in a second report string.
4. Finally, all records underwent another manual annotation process by a technical expert focusing mainly on qualitative signal characteristics.

### Preprocessing

The waveform files were converted from the original proprietary format into a binary format with 16 bit precision at a resolution of 1 *μ*V/LSB. The signals underwent minor processing to remove spikes from switch - on and switch- off processes of the devices, which were found at the beginning and the end of some recordings, and were upsampled to 500 Hz by resampling. For the user’s convenience, we also release a downsampled version of the waveform data at a sampling frequency of 100 Hz.

With the acquisition of the original database from Schiller AG, the full usage rights were transferred to the PTB. The Institutional Ethics Committee approved the publication of the anonymous data in an open-access database (PTB-2020-1). ECGs and patients are identified by unique identifiers. Instead of date of birth we report the age of the patient in years at the time of data collection as calculated using the ECG date. For patients with ECGs taken at an age of 90 or older, age is set to 300 years to comply with Health Insurance Portability and Accountability Act (HIPAA) standards. All ECG dates were shifted by a random offset for each patient while preserving time differences between multiple recordings. The names of validating cardiologists and nurses and recording site (hospital etc.) of the recording were pseudonymized and replaced by unique identifiers. The original data contained implausible height values for some patients. We decided to remove the height values for patients where the body-mass-index calculated from height and weight was larger than 40.

The ECG data was annotated using a codebook (SCP-ECG v0.4 (Annex B)) of ECG statements that preceded the current SCP-ECG standard^12^. All annotations were converted into SCP-ECG statements by accounting for the minor modifications that occurred between the release of the codebook and the publication of the final standard.

## Data Records

The data is composed of the ECG signal waveform data and additional metadata that comprises, most importantly, ECG statements in accordance with the SCP-ECG standard^12^. This section describes the components of the released data repository^5^ in detail and is organized as follows: In *Waveform Data*, we describe how the ECG signal waveform data is stored. *Metadata* describes the heart of *PTB-XL* including all information attached to each record.

### Waveform Data

For the user’s convenience, we provide waveform data in the WaveForm DataBase (WFDB) format as proposed by PhysioNet (https://physionet.org/about/software/) that has developed into an de-facto standard for the distribution of physiological signal data. In particular, there exist WFDB-parsers for a large number of frequently used programming languages such as C, Python, MATLAB and Java. In addition, the WFDB library also provides conversion routines to other frequently used data formats such as the European Data Format (edf). We stress that the original 16 bit binary data obtained after the conversion from the proprietary file format used by the ECG devices remained unchanged during this process. The WFDB-format only allows for a structured way of accessing the data that includes all required signal-specific metadata, such as channel names or conversion to physical units. In the WFDB-format every ECG is represented by a tuple of two files, a dat-file containing the binary raw data and a corresponding header file with same name and hea-extension. We provide both the original data sampled at 500 Hz as well as a downsampled version at 100 Hz that are stored in respective output folders records100 and records500.

### Metadata

The WFDB-format does not provide a standardized way of storing signal-specific metadata. For easy accessibility, we provide the metadata for all ECG records as a table in comma-separated value (csv) format in ptbxl_database.csv containing 28 columns, which can be easily accessed by using existing libraries in all common programming languages. Table 2 gives an overview of the columns provided in this table.

There are in total 21837 signals from 18885 patients. Figure 2 gives an graphical overview of the temporally ordered dataset in terms of populated fields, where black pixels indicating populated fields and white pixels indicating missing values. Please note how the data acquisition process changed over time, i.e. in the beginning of this study physiological data such as height and weight were gathered more often (mostly diagnostic reports written in English). Also note that towards the end of the study, the fraction of automated reports increases.

A detailed breakdown in terms of number of ECGs per patient is given in Table 3. In particular, there are $2127$ patients for which multiple ECGs available that could be used for longitudinal studies. The rest of this section is organized according to the sections headings in Table 2.

#### Identifiers

Each ECG record is identified by a unique ID (ecg_id) and the corresponding patient is encoded by a patient ID (patient_id). The path to the corresponding waveform data is stored in filename_lr (100 Hz) and filename_hr (500 Hz).

#### General Metadata

This section covers demographic data and general recording metadata contained in *PTB-XL*. Demographic data includes age, sex (52% male and 48% female), height (values set for 31.98% of records) and weight (values set for 43.18% of records). The age denotes the patient’s age at the time of the ECG recording. The distributions of age, height, and weight across the whole dataset are shown in Fig. 3. The median age is 62 with interquantile range (IQR) of 22 with minimum age of 0 and maximum age of 95. The median height and weight are 166 and 70 with IQRs of 14 and 20 respectively.

The general recording metadata comprises nurse, site, device and recording_date. Both nurse and site are published in pseudonymized form, where in total there are $12$ unique nurses across $51$ sites, i.e. the location where the ECG was recorded, and recorded using $11$ different types of devices. The field recording_date is encoded as YYYY-MM-DD hh:mm:ss.

#### ECG Statements

This section introduces the ECG statements as the core component of *PTB-XL*. It is organized as follows: First, we introduce the most important fields, namely report and scp_codes. Afterwards, heart_axis, infarction_stadium1 and infarction_stadium2 are discussed. Finally, we introduce the fields validated_by, second_opinion, initial_autogenerated_report and validated_by_human that are important for the technical validation of the annotation data.

**report** **and** **scp_codes:** The original ECG report is given as string in the report-column and is written in 70.89% German, 27.9% English, and 1.21% Swedish. The ECG report string was converted into structured sets of SCP-ECG statements as described in Methods. All information related to the used annotation scheme is stored in a dedicated table scp_statements.csv that was enriched with additional side-information, see *Conversion to other Annotation Standards* in *Usage Notes* for further details.

There are $71$ unique SCP-ECG statements used in the dataset. We categorize them by assigning each statement to one or more of the following categories: *diagnostic*, *form* and *rhythm* statements. There are 44 different diagnostic statements, 19 different form statements describing the form of the ECG signal, where 4 statements for diagnostic and form coincide, 12 different non-overlapping rhythm statements describing the cardiac rhythm (Fig. 4 gives an overview as a Venn-diagram of the proposed categories and their overlap). In addition, for all diagnostic statements, a likelihood information was extracted based on certain keywords in the ECG report, see Table 4 for details which is based on^7^. The likelihood ranges from 0 to 100 conveying the certainty the cardiologist (if the diagnosing cardiologist is very certain about a statement). For form and rhythm statements or in cases where no likelihood information was available, the corresponding likelihood was set to zero. The likelihood information is potentially interesting to account for the non-binary nature of diagnosis statements in real-world data. The SCP statements are presented as a unsorted dictionary (i.e. particular ordering of the statements within the dictionary does not follow any priority) of SCP-ECG statements in the scp_codes-column, where the key relates to the statement itself and the value relates to the likelihood.

Finally, for diagnostic statements we provide a hierarchy of superclasses and subclasses that can be used to train classification algorithms on a set of broader categories instead of the original fine-grained diagnostic labels, see Table 5 for a definition of the acronyms and Fig. 1 for graphical overview of the whole dataset. Tables summarizing the distribution of diagnostic, form and rhythm statements can be found in Tables 6, 7 and 8 respectively, where the first column indicates the acronym associated with the statement (Table 5 for description of acronyms), the second column reflects the number of records (ordered ascending) and the third column gives a short description for each statement. In addition for Table 6 we provide two additional columns indicating the proposed super- and subclass. If we aggregate the diagnostic statements according to superclasses and subclasses using the mapping as described above and in Table 5, the distribution of diagnostic superclass statements assumes the form shown in the uppermost panel in Fig. 5. Particular mentioning deserves the large number of healthy patients that are typically underrepresented in most ECG datasets that are, however, crucial for the development of ECG classification algorithms. Figure 5 shows the distribution of subclasses for a given diagnostic superclass.

In summary, we provide six sets of annotations with different levels of granularity, namely raw (all statements together), diagnostic, diagnostic superclass, diagnostic subclass statements, form and rhythm statements. Depending on granularity, a different number of statements per ECG record is available. A detailed breakdown in terms of number of statements in each level per ECG signal is given in Table 9. For example, there are 410 samples for which no diagnostic statement is given, which are mainly pacemaker ECGs.

**heart_axis**, **infarction_stadium1** **and** **infarction_stadium2:** The column heart_axis was automatically extracted from the ECG report and is set for 61.05% of the records. It represents the heart’s electrical axis in the Cabrera system. Table 10 shows the distribution, the acronyms and the respective descriptions for entries in the column heart_axis.

In case of myocardial infarction, potentially multiple entries for infarction stadium (infarction_stadium and infarction_stadium2) were extracted from the report string. Table 11 shows the respective distributions in addition to a short description, see^7^ for further details. In particular, we distinguish also intermediate stages “stadium I-II” and “stadium II-III” in addition to the conventionally used infarction stages I, II, and III.

**validated_by** **and** **second_opinion:** The validated_by-column provides the identifier of the cardiologist who performed the initial annotation. The column second_opinion is set to true for records, where a second opinion is available and the corresponding report string is appended to report with a preceding “Edit:”. The column initial_autogenerated_report is set to true for all records, where the report string ended with “*unbestätigter Bericht*’” indicating that the initial report string was generated by an ECG device, as described in *Data Acquisition*. Unfortunately, there is no precise record of the ECGs that underwent the second validation. For this reason, we store a conservative estimate if the record was validated by a human cardiologist in the column validated_by_human. It is set to true for all records, where validated_by is set, or initial_autogenerated_report is false, or second_opinion is true, see* Quality Assessment for Annotation Data (ECG Statements)* in *Technical Validation* for more details.

#### Signal Metadata

As additional metadata that might potentially be of future use, the signal quality was quantified by a different person with long technical expertise in ECG devices and signals, who went through the whole dataset and annotated the records with respect to signal characteristics such as noise (static_noise and burst_noise), baseline drifts baseline_drift and other artifacts such as electrodes_problems. In addition to these technical signal characteristics, we provide extra_beats for counting extra systoles which is set for 8.95% of records and pacemaker for signal patterns indicating an active pacemaker (for 1.34% of records).

Possible findings in each of the different categories are reported as string without a regular syntax. Overall, these reports represent a very rich source of additional information. The most basic use of these fields is to filter for data of a particularly high quality by excluding all records with non-empty values in the columns mentioned above. We refer to *Quality Assessment for Waveform Data* in *Technical Validation* for a summary of the signal quality in terms of the provided annotations.

#### Cross-validation Folds

For comparability of machine learning algorithms trained on *PTB-XL*, we provide fold assignments (strat_fold) for all ECG records that can be used to implement recommended train-test splits. The incentive to use stratified sampling is to reduce bias and variance of score estimations, see^17^. In addition, it leads to a test set distribution for holdout evaluation that mimics the training set distribution as closely as possible to disentangle aspects of covariate shift/dataset shift from the evaluation procedure. We extend existing multilabel stratification methods from the literature to achieve a balanced label while additionally providing two distinguished folds with a particularly high label quality. During this process, each record is assigned to one of ten folds, where the tenth fold is intended to be used for holdout set evaluation and the penultimate ninth fold is supposed to be used as validation set, see *Prediction Tasks and Train-Test-Splits for ML Algorithms* in *Usage Notes* for a more detailed description. The fold assignment always respects the underlying patient assignments. This avoids data leakage arising from having ECG signals from the same patient in different folds. In detail, the fold assignment proceeds as follows:

The proposed procedure extends existing stratified sampling methods from the literature^18^ by accounting for sampling based on patients and by optionally incorporating quality constraints for certain folds. To achieve not only a balanced label distribution but also a balanced age and sex distribution, we do not only incorporate all ECG statements but also sex and age (in five bins each covering 20 years). All ECG statements, sex and age for a given patient are appended into a single list with potentially non-unique entries to ensure sampling based on patients. Then the labels are distributed label-by-label as proposed^18^, starting with the least populated label within the remaining records. Patients with ECG records that are annotated with this label are subsequently distributed onto the folds. If there is a unique fold that is in most need of the given label, all ECGs of the patient that is currently under consideration are assigned to this fold. In case of a tie, the assignment proceeds by trying to balance the overall sizes of the candidate folds.

During this process, we keep track of the quality of the ECG annotations. A patient is considered *clean* if for all corresponding ECGs validated_by_human is set to true. When assigning ECGs from a patient that does not carry this flag, we exclude the ninth and tenth fold from the set of folds the samples can be assigned to. As the dataset and in particular the ratio of *clean* vs. non-*clean* patients is large enough, the sampling procedure still leads to a label distribution in the *clean* folds that still approximates the overall distribution of labels and sexes in the dataset very well, see Fig. 6.

We believe that this procedure is of general interest for multi-label datasets with multiple records per patient and, in particular in the current context, for exploring the impact of different stratification methods. For the fold assignments in strat_fold, we based the stratification on all available ECG statements but it might also conceivable to consider just subsets of labels, such as all diagnostic statements. To allow a simple exploration of these issues, we provide a Python implementation of the stratification method in the Supplementary Material.

## Technical Validation

### Quality Assessment for Waveform Data

Since we present the waveform data in its original (binary) form without any modifications (apart from saving it in WFDB-format), we expect a lot of variability with respect to recording noise and several artifacts. For this purpose we summarize the results of the technical validation of the signal data by an technical expert briefly. The signal quality was quantified by a person with technical expertise according to the following categories:

- baseline_drift for global drifts in 7.36% of the signal.
- static_noise for noisy signals and burst_noise for noise peaks, set for 14.94% and 2.81% of records retrospectively.
- electrodes_problems for individual problems with electrodes (0.14% of records).

In total 77.01% of the signal data are of highest quality in the sense of missing annotation in the signal quality metadata. At this point we would like to stress again that the different quality levels reflect the range of different quality levels of ECG data in real-world data and have to be seen as one of the particular strengths of the dataset. This dataset contains a realistic distribution of data quality in clinical practice and is an invaluable source for properly assessing the performance of ML algorithms in the sense of the robustness against changes in the environmental conditions or against various imperfections in the input data.

### Quality Assessment for Annotation Data (ECG Statements)

As already mentioned in *ECG Statements*, it has not been possible to retrospectively reconstruct the labeling process in all cases. In some cases the validating cardiologist (validated_by-column) was left empty even though an automatically created initial ECG report (autogenerated_initial_report) was validated by a human cardiologist. In addition, there is no precise record of those ECGs that went through the second human validation step. Before submission, we randomly selected a subset of recordings from our proposed test set via strati fied sampling (as described in *Crossvalidation Folds*) and had them reviewed by another independent cardiologist (Author FIL). These examinations confirmed the annotations.

Due to missing information about this process, we can only conservatively estimate that set of ECGs that were potentially only automatically annotated. Therefore, we set validated_by_human to false for the set of automatically annotated ECGs (initial_autogenerated_report=True) with empty validated_by-column and second_opinion=False. The precise fractions are as follows:

- 73.7% validated_by_human=True
  - 56.9% validated_by is given
  - 16.18% initial_autogenerated_report=False
  - 0.62% second_opinion is given
- 26.3% validated_by_human=False

This is to the best of our knowledge a very conservative estimate as a large fraction of the dataset went through the second validation step, but from our perspective the most transparent way of dealing with this missing metadata issue. Moreover, the second validation was not performed independently but as an validation of the first annotation. Unfortunately, there is no precise record of which diagnostic statements were changed during the final validation step. Therefore, even though most records were evaluated by two cardiologists (albeit not independently), one can only reasonably claim a single human validation.

To make best use of the available data, we decided to incorporate the information which ECGs certainly underwent human validation into the sampling process. To this end, we construct the fold assignment process in such a way that the tenth fold only contains only ECGs that certainly underwent a human validation. This allows to use the tenth fold as a reliable test set with best available label quality for a simple hold-out validation. This is described in detail in *Prediction Tasks and Train-Test-Splits for ML Algorithms* in *Usage Notes*.

## Usage Notes

In this section, we provide instructions on how to use *PTB-XL* to train and validate automatic ECG interpretation algorithms. To this end, we first explain how to convert to other standards than SCP in *Conversion to other Annotation Standards*, afterwards we explain in *Prediction Tasks and Train-Test-Splits for ML Algorithms* how the proposed cross-validation folds are supposed to be used for a reliable benchmarking of machine learning algorithms on this dataset and outline possible prediction tasks on the dataset. Finally, in *Example Code* we provide a basic code example in Python that illustrates how to load waveform data and metadata for further processing and provide directions for further analysis.

### Conversion to other Annotation Standards

As already mentioned in *ECG Statements*, besides our proposed SCP standard, we also provide the possibility of transition to other standards such as the scheme put forward by the American Heart Association^19^. For this purpose and the user’s convenience our repository also provides SCP_labelmap.csv with further information, see *ECG Statements* for details on the used SCP-ECG statements.

Table 12 gives a detailed description of the table scp_statements.csv. The first column serves as index with SCP statement acronym, the second, eighth and ninth column (description, Statement Category, SCP-ECG Statement Description) describes the respective acronym. The third, fourth and fifth column (diagnostic, form and rhythm) indicate to which broad category each index belongs to. The sixth and seventh column (diagnostic_class and diagnostic_subclass) describes our proposed hierarchical organization of diagnostic statements, see *ECG Statements* for additional information on the latter two properties.

The latter three columns of Table 12 provide cross-references to other popular ECG annotation systems as provided on the SCP-ECG homepage (http://webimatics.univ-lyon1.fr/scp-ecg/), namely: AHA aECG REFID, CDISC and DICOM. In *Example Code*, we provide example Python code for using scp_statements.csv appropriately.

### Prediction Tasks and Train-Test-Splits for ML Algorithms

The *PTB-XL* dataset represents a very rich resource for the training and the evaluation of ECG analysis algorithms. Whereas a comprehensive discussion of possible prediction tasks that can be investigated based on the dataset is clearly beyond the format of this data descriptor, we still find it worthwhile sketching possible future direction. The most obvious tasks are prediction tasks that try to infer different subsets of ECG statements from the ECG record. These tasks can typically be framed as multi-label classification problems. Although a thorough description of proposed evaluation metrics would go beyond of the scope of this manuscript, we highly recommend macro-averaged and threshold-free metrics, such as the macro-averaged area under the receiver operating curve (AUROC). Micro-averaged metrics would overrepresent highly populated classes, whose distribution just reflects the data collection process rather than the statistical distribution of the different pathologies in the population. The large number of more than 2000 patients with multiple ECGs potentially allows to develop prediction models for future cardiac conditions or their progression from previously collected ECGs. Beyond ECG statement prediction, the dataset allows for age/sex inference from the raw ECG record and to develop ECG quality assessment algorithms based on the signal quality annotation. Finally, the provided likelihoods for diagnostic statements can be used to study possible relations between prediction uncertainty compared to human uncertainty assessments.

For comparability of machine learning algorithms trained on *PTB-XL*, we provide recommended train-test splits in the form of assignments of the record to one of ten cross-validation folds. We propose to use the tenth fold, which is ensured to contain only ECGs that have certainly be validated by at least one human cardiologist and are therefore presumably of highest label quality, to separate a test set that is only used for the final performance evaluation of a proposed algorithm. The remaining nine folds can be used as training and validation set and split at one’s own discretion potentially utilizing the recommended fold assignments. As the ninth and the tenth fold satisfy the same quality criteria, we recommend to use the ninth fold as validation set.

### Example Code

In Fig. 7, we provide a basic code example in Python for loading both waveform and metadata, aggregating the diagnostic labels based on the proposed diagnostic superclasses and split data into train and test set using the provided crossvalidation folds. The two main resulting objects are the raw signal data (as a numpy array of shape 1000 × 12 for the case of 100 Hz data) loaded with wfdb as a numpy array as described in *Waveform Data* and the annotation data from ptbxl_database.csv as a pandas dataframe with 26 columns as described in *Metadata*. In addition, we illustrate, how to apply the the provided mapping of individual diagnostic statements to diagnostic superclass mapping as introduced in *ECG Statements* and described in *Conversion to other Annotation Standards* which consists of loading scp_statements.csv, selecting for diagnostic and creating multi-label lists by applying diagnostic_superclass given the index. Finally, we apply the suggested split into train and test as described in *Prediction Tasks and Train-Test-Splits for ML Algorithms*.

After the raw data has been loaded, there are different possible directions for futher analysis. First of all, there are dedicated packages such as BioSPPy (https://github.com/PIA-Group/BioSPPy) that allow to extract ECG-specific features such as R-peaks. Such derived features or the raw signals themselves can then be analyzed using classical machine learning algorithms as provided for example by scikit-learn (https://scikit-learn.org) or popular deep learning frameworks such as TensorFlow (https://www.tensorflow.org) or PyTorch (https://pytorch.org).

## Supplementary information

Supplementary File 1

## Data Availability

The code for dataset preparation is not intended to be released as it does not entail any potential for reusability. We provide the stratified sampling routine in Supplementary File 1 to allow users to create stratification folds based on user-defined preferences.
