# Supplementary material for: PTB-XL, a large publicly available electrocardiography dataset
Source: Sci Data. 2020 May 25;7:154. doi: 10.1038/s41597-020-0495-6 (PMC7248071; doi:10.1038/s41597-020-0495-6)
Supplement: Supplementary file 1 [file 41597_2020_495_MOESM1_ESM.pdf]

# Supplementary File 1

## Python code for stratified sampling

```
import numpy as np
from tqdm import tqdm

def stratify(data, classes, ratios, qualities, ecgs_per_patient,
    nr_clean_folds=1):
    """Stratifying procedure. Modified from https://vict0rs.ch
    /2018/05/24/sample-multilabel-dataset/ (based on Sechidis 2011)

    data is a list of lists: a list of labels, for each sample.
        Each sample's labels should be ints, if they are one-hot
    encoded, use one_hot=True

    classes is the list of classes each label can take

    ratios is a list, summing to 1, of how the dataset should be
    split

    qualities: quality per entry (only >0 can be assigned to clean
     folds; 4 will always be assigned to final fold)

    ecgs_per_patient: list with number of ecgs per sample

    nr_clean_folds: the last nr_clean_folds can only take clean
    entries

    """
    np.random.seed(0) # fix the random seed

    # data is now always a list of lists; len(data) is the number
    of patients; data[i] is the list of all labels for patient i (
    possibly multiple identical entries)

    #size is the number of ecgs
    size = np.sum(ecgs_per_patient)

    # Organize data per label: for each label l, per_label_data[l]
     contains the list of patients
    # in data which have this label (potentially multiple
    identical entries)
    per_label_data = {c: [] for c in classes}
    for i, d in enumerate(data):
        for l in d:
            per_label_data[l].append(i)

    # In order not to compute lengths each time, they are tracked
    here.
    subset_sizes = [r * size for r in ratios] #list of
    subset_sizes in terms of ecgs
    per_label_subset_sizes = { c: [r * len(per_label_data[c]) for
    r in ratios] for c in classes } #dictionary with label: list of
     subset sizes in terms of patients
```

```
# For each subset we want, the set of sample-ids which should
end up in it
stratified_data_ids = [set() for _ in range(len(ratios))] #
initialize empty

# For each sample in the data set
print("Assigning patients to folds...")
size_prev=size+1 #just for output
while size > 0:
    if(int(size_prev/1000) > int(size/1000)):
        print("Remaining patients/ecgs to distribute:",size,"
non-empty labels:", np.sum([1 for l, label_data in
per_label_data.items() if len(label_data)>0]))
    size_prev=size
    # Compute |Di|
    lengths = {
        l: len(label_data)
        for l, label_data in per_label_data.items()
    } #dictionary label: number of ecgs with this label that
have not been assigned to a fold yet
    try:
        # Find label of smallest |Di|
        label = min({k: v for k, v in lengths.items() if v >
0}, key=lengths.get)
    except ValueError:
        # If the dictionary in ‘min‘ is empty we get a Value
Error.
        # This can happen if there are unlabeled samples.
        # In this case, ‘size‘ would be > 0 but only samples
without label would remain.
        # "No label" could be a class in itself: it’s up to
you to format your data accordingly.
        break
    # For each patient with label ‘label‘ get patient and
corresponding counts
    unique_samples, unique_counts = np.unique(per_label_data[
label],return_counts=True)
    idxs_sorted = np.argsort(unique_counts, kind='stable')
[::-1]
    unique_samples = unique_samples[idxs_sorted] # this is a
list of all patient ids with this label sort by size descending
    unique_counts =  unique_counts[idxs_sorted] # these are
the corresponding counts

    # loop through all patient ids with this label
    for current_id, current_count in zip(unique_samples,
unique_counts):

        subset_sizes_for_label = per_label_subset_sizes[label]
 #current subset sizes for the chosen label

        #if quality is bad remove clean folds (i.e. sample
cannot be assigned to clean folds)
        if(qualities[current_id] < 1):
            subset_sizes_for_label = subset_sizes_for_label[:
len(ratios)-nr_clean_folds]
```

```
        # Find argmax clj i.e. subset in greatest need of the
current label
        largest_subsets = np.argwhere(subset_sizes_for_label
== np.amax(subset_sizes_for_label)).flatten()

        # if there is a single best choice: assign it
        if len(largest_subsets) == 1:
            subset = largest_subsets[0]
        # If there is more than one such subset, find the one
in greatest need of any label
        else:
            largest_subsets2 = np.argwhere(np.array(
subset_sizes)[largest_subsets] == np.amax(np.array(subset_sizes
)[largest_subsets])).flatten()
            subset = largest_subsets[np.random.choice(
largest_subsets2)]

        # Store the sample's id in the selected subset
        stratified_data_ids[subset].add(current_id)

        # There is current_count fewer samples to distribute
        size -= ecgs_per_patient[current_id]
        # The selected subset needs current_count fewer
samples
        subset_sizes[subset] -= ecgs_per_patient[current_id]

        # In the selected subset, there is one more example
for each label
        # the current sample has
        for l in data[current_id]:
            per_label_subset_sizes[l][subset] -= 1

        # Remove the sample from the dataset, meaning from all
 per_label dataset created
        for x in per_label_data.keys():
            per_label_data[x] = [y for y in per_label_data[x]
if y!=current_id]

# Create the stratified dataset as a list of subsets, each
containing the orginal labels
stratified_data_ids = [sorted(strat) for strat in
stratified_data_ids]
stratified_data = [
    [data[i] for i in strat] for strat in stratified_data_ids
]

# Return both the stratified indexes, to be used to sample the
 `features` associated with your labels
# And the stratified labels dataset

return stratified_data_ids, stratified_data
```
